# Enhanced Stability and Detection Range of Microbial Electrochemical Biotoxicity Sensor by Polydopamine Encapsulation

**DOI:** 10.3390/bios14080365

**Published:** 2024-07-26

**Authors:** Zengfu Guan, Jiaguo Yan, Haiyuan Yan, Bin Li, Lei Guo, Qiang Sun, Tie Geng, Xiaoxuan Guo, Lidong Liu, Wenqing Yan, Xin Wang

**Affiliations:** 1 Oilfield Chemicals Division, China Oilfield Services Limited (COSL), Tianjin 300450, China; guanzf@cosl.com.cn (Z.G.); yanhy7@cosl.com.cn (H.Y.); libin21@cosl.com.cn (B.L.); guolei5@cosl.com.cn (L.G.); sunqiang7@cosl.com.cn (Q.S.); gengtie@cosl.com.cn (T.G.); guoxx6@cosl.com.cn (X.G.); liuld2@cosl.com.cn (L.L.); 2 Tianjin Marine Petroleum Environmental and Reservoir Low-Damage Drilling Fluid Enterprise Key Laboratory, Tianjin 300450, China; 3 MOE Key Laboratory of Pollution Processes and Environmental Criteria, Tianjin Key Laboratory of Environmental Remediation and Pollution Control, College of Environmental Science and Engineering, Nankai University, No. 38 Tongyan Road, Jinnan, Tianjin 300350, China; xinwang1@nankai.edu.cn

**Keywords:** microbial electrochemical system, polydopamine, toxicity sensor, electroactive biofilm, heavy metals ion detection

## Abstract

With the rapid development of modern industry, it is urgently needed to measure the biotoxicity of complex chemicals. Microbial electrochemical biotoxicity sensors are an attractive technology; however, their application is usually limited by their stability and reusability after measurements. Here, we improve their performance by encapsulating the electroactive biofilm with polydopamine (PDA), and we evaluate the improvement by different concentrations of heavy metal ions (Cu^2+^, Ag^+^, and Fe^3+^) in terms of inhibition ratio (*IR*) and durability. Results indicate that the PDA-encapsulated sensor exhibits a more significant detection concentration than the control group, with a 3-fold increase for Cu^2+^ and a 1.5-fold increase for Ag^+^. Moreover, it achieves 15 more continuous toxicity tests than the control group, maintaining high electrochemical activity even after continuous toxicity impacts. Images from a confocal laser scanning microscope reveal that the PDA encapsulation protects the activity of the electroactive biofilm. The study, thus, demonstrates that PDA encapsulation is efficacious in improving the performance of microbial electrochemical biotoxicity sensors, which can extend its application to more complex media.

## 1. Introduction

With the acceleration of industrialization, the variety and quantity of chemical substances have increased sharply, posing a potential threat to the ecological system and human health. Biotoxicity detection, a crucial step in assessing these chemicals’ safety, has become increasingly important [1,2]. Many methods are available for this purpose, such as classical in vivo animal biotoxicity detection involving mice [3], zebrafish [4], and brine shrimp [5]. Although in vivo animal experiments can provide the most accurate feedback on biological toxicity, they often suffer from drawbacks such as long detection times and poor reproducibility, along with violating scientific ethics [6]. As a result, faster and more accurate toxicity testing methods have emerged, including bioluminescent bacteria assays [7], thermistor-based methods [8], electrochemical cyclic voltammetry sensors [9], whole-cell biosensors [10], and molecular biology-based biosensors [11], among others.

Among these, the microbial electrochemical system-based biotoxicity sensors, which employ electroactive biofilms as the sensing element, are promising due to their rapid response, cost-effectiveness, and relatively higher reproducibility. For example, microbial electrolysis cell (MEC) sensors can achieve real-time monitoring of toxins’ toxicity by monitoring changes in electric current [12,13], thereby significantly reducing the detection period and enhancing emergency response capabilities. However, the activity of the electroactive biofilm is susceptible to external environmental fluctuations, whereas high concentrations of toxicants and frequent detections may cause irreversible damage to the biofilm, resulting in a technical bottleneck to its widespread application [14,15]. It is, therefore, imperative to improve the stability of the electroactive biofilm.

One of the most effective ways to protect the electroactive biofilm is polymer encapsulation. As a naturally occurring high molecular weight polymer with excellent biocompatibility, polydopamine (PDA) is a promising encapsulant. Due to its outstanding adhesive properties, it can form a protective layer over the electroactive biofilm, preventing external adverse factors from interfering with the microbes and electrodes [16,17]. It has been demonstrated that an electroactive biofilm encapsulated with PDA was resistant to extremely low pH, recovering after 30 min of exposure to shocks of pH 0.5 and 1.5 [18]. These results provide an inspiration for preparing a highly resistant biofilm.

Here, we used PDA encapsulation to improve the stability and reusability of a microbial electrochemical biotoxicity sensor. The PDA-encapsulated electroactive biofilm-based microbial electrochemical toxicity sensor was tested for the toxicity of heavy metal ions. Compared with the sensor without the PDA package, the standard curve and *EC50* were calculated to show a higher detection range and multiplexing effect, indicating that the microbial electrochemical toxicity sensor in the PDA package was more feasible for commercialization.

## 2. Material and Methods

### 2.1. The MEC Sensor Construction

The MEC-type sensor consisted of a dual-electrode system, with a cylindrical carbon brush as the working electrode, where the electroactive biofilm was placed. The carbon brush had a length of 7 cm and a diameter of 4 cm. The counter electrode was made of stainless steel mesh (type 304N, 60 mesh) in a hollow cylindrical shape with a diameter of 4.5 cm and a height of 7 cm, allowing the carbon brush to be fixed in the center of the stainless steel mesh cylinder. The reactor container was made of a resin bottle with an effective volume of approximately 120 mL, having an inner diameter of 4.5 cm and a height of 8 cm. A small hole, to be sealed with a rubber stopper, was made at the top of the container for injecting toxins. MEC sensors were inoculated with a mixed effluent of acetate-fed MFCs in our laboratory. A direct current voltage of 0.7 V was applied to the MEC-type sensor, and the current data was recorded at a regular frequency of once every 12 s. The culture medium consisted of a 100 mL volume composed of 1 g/L sodium acetate in 50 mM phosphate buffer solution (PBS: 4.576 g/L Na_2_HPO_4_, 2.132 g/L NaH_2_PO_4_, 0.31 g/L NH_4_Cl, 0.13 g/L KCl, pH = 7.2–7.4), 10 mL/L mineral solution, and 15 mL/L vitamin solution; composition of both solutions was described in the previous study [19]. A volume of 20 mL culture medium was reserved for the toxicity experiment.

### 2.2. Encapsulation of the Electroactive Biofilm with PDA and the Heavy Metal Ion Shock

When the electroactive biofilm on the working electrode of the MEC sensors matured, approximately after five cycles, the current stabilized at around 20 mA. Dopamine was dissolved in a Tris-HCl buffer solution (10 mmol/L, pH = 8.5) to obtain a dopamine solution at a concentration of 2 g/L. Subsequently, the working electrode was removed and immersed in the dopamine solution for 30 min, allowing dopamine to polymerize on the surface of the anodic biofilm to achieve an anodic electroactive biofilm encapsulated with PDA [20]. The electrode was then rinsed with PBS to remove any residual alkaline solution.

Various concentrations (10 ppm, 30 ppm, 50 ppm, 70 ppm, 100 ppm, 150 ppm, 200 ppm, 250 ppm, and 300 ppm) of three different heavy metal ions, including Cu^2+^, Ag^+^, and Fe^3+^, were used in the toxicity tests. During the intermittent toxicity experiments, 1 mL of the heavy metal ion solution was added, respectively, through the small hole reserved at the top of the sensor. Subsequently, changes in the electric current were noted after 10-min intervals. Once the current reached a stable level, the culture medium was replaced to prevent the toxins from having a long-term impact on the electroactive biofilm of the sensor. Each test was conducted in duplicate for reproducibility.

The response of the sensor to the impact of the heavy metal ions was recorded as the inhibition ratio (*IR*), calculated as *IR* = (*I_max_* − *I_min_*)/*I_sta_*, where *I_max_* is the maximum current value within ten minutes after adding the toxin, *I_min_* is the minimum current value within ten minutes after adding the toxin, and *I_sta_* is the stable current value before adding the toxin. Subsequently, the correlation between the concentration of the heavy metal ions and the *IR* was plotted and fitted to a linear model to calculate the limit of detection (*LOD*), 3*δ*/*K*, and *EC50*. Here, *δ* represents the standard deviation of the *IR* calculated from blank samples (with a toxin concentration of 0 ppm), *K* is the slope of the fitted line between the heavy metal ion concentration and *IR*, and *EC50* is the concentration of the heavy metal ions corresponding to *IR* = 50%, calculated based on the fitted line.

For the continuous toxicity experiments, 1 mL of 30 ppm Cu^2+^ solution was added through the small hole reserved at the top of the sensor, and changes in the current were monitored for 10 min. Once the current reached a stable level, another 1 mL of the 30 ppm Cu^2+^ solution was added. This process was repeated 20 times to assess the impact of repeated exposure of the sensor to toxic substances. Furthermore, the test was repeated for reproducibility. In the continuous toxicity experiments, the effect of the toxin on the sensor is represented by the percentage of current, calculated as the current percentage, *I*/*I_sta_*, where *I* is the current value changing over time, and *I_sta_* is the stable current value before adding the toxin.

### 2.3. Biofilm Topography and Electrochemical Analysis

To observe the electroactive biofilm before and after PDA encapsulation, we used a Field Emission Scanning Electron Microscope (FE-SEM, JSM-7800F, JEOL, Akishima, Japan). The samples were prepared by using sterile scissors to cut off the carbon brush bristles. These were then treated with a 2.5% glutaraldehyde fixative solution for 12 h and dehydrated for 10 min using a series of ethanol solutions with different concentration gradients (30%, 50%, 70%, 90%, and 100%), respectively [21].

To capture the viability of the electroactive biofilm before and after the continuous toxicity experiments, we used a Confocal Laser Scanning Microscope (CLSM, LSM880 with Airyscan, Zeiss, Oberkochen, Germany). The samples were prepared similarly to those of the SEM by using sterile scissors to cut off the carbon brush bristles. However, for the CLMS, the samples were stained with the LIVE/DEAD BacLight Bacterial Viability Kit (L13152, ThermoFisher Scientific Inc., Waltham, MA, USA) for 20 min. The stained samples were rinsed off with 50 mM PBS to remove any excess dye. A previously reported photography method was followed to study the samples [22].

Cyclic voltammetry (CV) was used to compare the electroactivity of the sensor before and after the continuous toxicity experiments. The CV tests were conducted on an electrochemical workstation (CHI 1000C, CH Instruments, Shanghai, China) in a potential range of −0.8 V to 0.2 V at a scan rate of 10 mV/s. An Ag/AgCl electrode was used as the reference electrode (3 M KCl, +0.197 V vs. SHE). Moreover, each scan consisted of two cycles. The first derivative of the CV (DCV) was derived from the CV plot to analyze changes in the redox activity of the electroactive biofilm before and after the continuous toxicity experiments.

## 3. Results and Discussion

### 3.1. Morphology of Biofilm and Baseline Current after PDA Encapsulation

**Figure 1:**
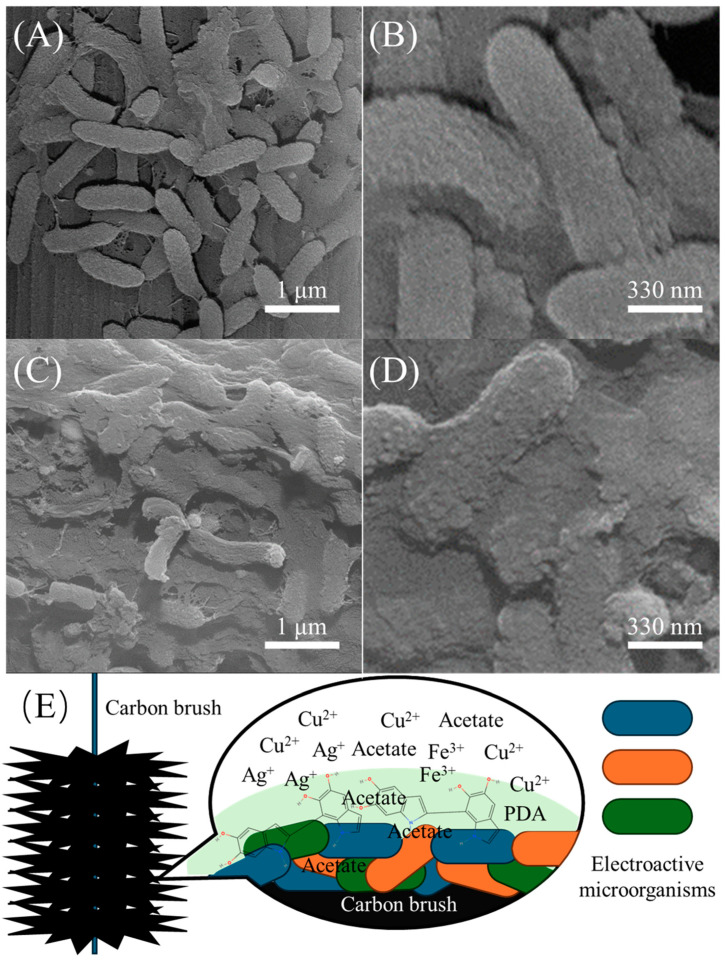
SEM images of the anode electroactive biofilms in controls (**A**,**B**), after PDA encapsulation (**C**,**D**), and a schematic of PDA encapsulation (**E**).

After stable operation of the microbial electrochemical system for five cycles, SEM images of the anodic electroactive biofilm were captured. In the control group without the PDA encapsulation (Figure 1A,B), the outlines of microbial cells are clearly visible, with filamentous connections between cells representing extracellular polymeric substances (EPS). The EPS provides protection and fixation for microbes to adhere firmly to the carbon brush surface [23]. However, in the experimental group with PDA encapsulation (Figure 1C,D), the outlines of the microbial cells are less distinct due to the presence of PDA on the surface of the biofilm, blurring the individual cell outlines. Furthermore, PDA has encapsulated the cells to merge, providing better protection to the electroactive biofilm [18], as shown in the schematic diagram (Figure 1E).

**Figure 2:**
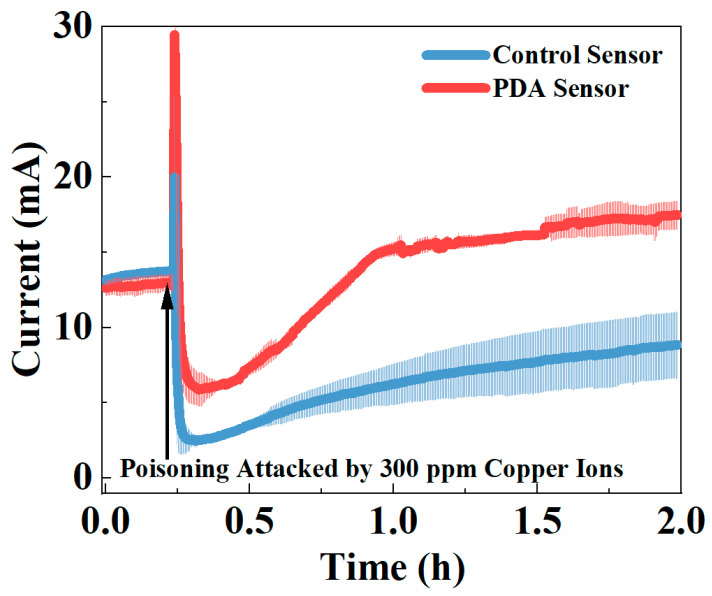
Current changes before and after adding 300 ppm copper ions in the control and PDA-encapsulated sensors.

After long-term operation, the current in the control group without PDA encapsulation stabilized at 13.5 ± 0.2 mA, slightly higher than the 12.8 ± 0.1 mA in the PDA encapsulation group (Figure 2). This difference may be due to the PDA coating on the surface of the electroactive biofilm affecting cellular uptake of organic compounds and extracellular electron transfer, but not severely. Upon the addition of 300 ppm Cu^2+^, both groups exhibited a trend of an initial increase, followed by a decrease and then a recovery in current. The initial increase in current is a stress response of the microbes. When exposed to the toxin, microbes initiate stress metabolism to protect their cells, leading to an increase in metabolic rate, reflected in the higher current in the electroactive biofilm [24]. Subsequently, due to the toxic effects of Cu^2+^, microbial metabolic activity is inhibited, and some cells may even die, resulting in a decrease in current [25]. The lowest current for the PDA encapsulation sensor was 5.9 ± 1.1 mA, more than twice the 2.5 ± 0.4 mA of the control group without PDA, indicating the protective effect of PDA on the electroactive biofilm. Notably, the protective effect of PDA on the electroactive biofilm was also evident in the recovery phase of the sensor current after the toxic impact. As shown in Figure 2, after 1.5 h of the toxin addition, the sensor with PDA encapsulation exhibited a higher stable current of 17.2 ± 1.1 mA, surpassing the level before the toxicity experiment. Additionally, within the following fifteen minutes, the standard deviation of the average current of the sensor varied by only 0.12 mA. In contrast, the control group without PDA encapsulation maintained a current of 8.3 ± 2.2 mA after 1.5 h of the toxin addition, only recovering to 61% of the pre-experiment current.

By using 300 ppm Cu^2+^ as the highest concentration, the study demonstrates that even under extremely toxic conditions, the current of the PDA-encapsulated sensor could recover to the level before the experiment, establishing the protective effect of PDA on the sensor. Moreover, the fluctuating current trend, initially increasing and then decreasing during the toxicity experiment, also reveals the reason for using the *IR* = (*I_max_* − *I_min_*)/*I_sta_* to describe the strength of the toxins’ toxicity.

### 3.2. Sensor Responses to Different Metal Ions

**Figure 3:**
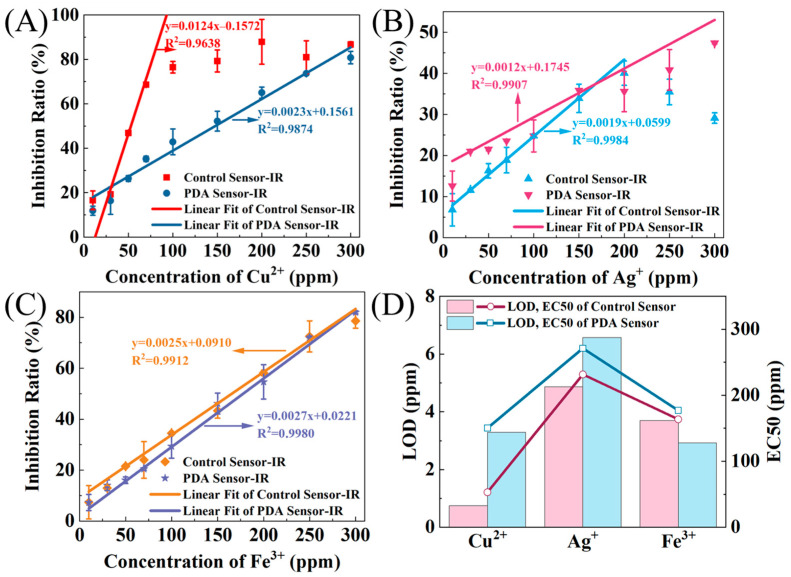
*IR* vs. heavy metal ion concentration (**A**) Cu^2+^, (**B**) Ag^+^, (**C**) Fe^3+^ curves (with linear fitting) and (**D**) *LOD* and *EC50* for the different metal ions of the control and PDA-encapsulated sensors.

The toxicity response of the two types of sensors, with and without PDA encapsulation, was compared for three different heavy metal ions in the concentration range of 10–300 ppm. The experimental results indicate a good linear relationship of the PDA-encapsulated sensor current response (*IR*) with the different heavy metal ions in the range of 10–300 ppm (Figure 3A–C). The representative linear equations for the *IR* versus Cu^2+^, Ag^+^, and Fe^3+^ concentrations are y = 0.0023x + 0.1561 (R^2^ = 0.9874), y = 0.0012x + 0.1745 (R^2^ = 0.9907), and y = 0.0027x + 0.0221 (R^2^ = 0.9980), respectively. However, for sensors without PDA encapsulation, the linear response range of the *IR* to the concentration of the different heavy metal ions is not consistent. For Cu^2+^, the linear response range turns out to be 10–100 ppm with the equation y = 0.0124x − 0.1572 (R^2^ = 0.9638) (Figure 3A). Beyond that, in the range of 100–300 ppm, the *IR* of the sensor fluctuates around 80%, indicating that the sensor without PDA encapsulation can only reach the detection limit of 100 ppm for Cu^2+^.

Similarly, the linear response range of the sensor without PDA encapsulation to Ag^+^ comes to around 10–200 ppm with the equation y = 0.0019x + 0.0599 (R^2^ = 0.9984) (Figure 3B). While, in the range of 200–300 ppm, a situation arises where, as the concentration of Ag^+^ increases, the *IR* decreases and falls below the *IR* of the PDA-encapsulated sensor. This behavior may be due to the irreversible changes in the community structure of the electroactive biofilm caused by Ag^+^ during the experimental process, enhancing the resistance of the electroactive biofilm. A previous study has demonstrated that using Pb^2+^ as a toxicant in MEC-type toxicity sensors could affect the community structure of the electroactive biofilm [26].

Surprisingly, the linear response range of the sensor without PDA encapsulation to Fe^3+^ is determined at 10–300 ppm, described by the equation y = 0.0025x + 0.0910 (R^2^ = 0.9912) (Figure 3C). As such, this result is not significantly different from the results obtained with the PDA-encapsulated sensor. It may be because the change in the current caused by Fe^3+^ is not due to a decrease in cell metabolic activity or induction of cell apoptosis. Studies have shown that iron can serve as an electron acceptor for extracellular electron transfer in electroactive microorganisms [27]. After adding Fe^3+^, the electron acceptor of the electroactive biofilm of the sensor changes from the electrode to Fe^3+^, leading to a decrease in the output current. Nevertheless, the extent of this change is only related to the concentration of Fe^3+^.

Although the mechanism by which Fe^3+^ and other heavy metal ions cause changes in current may differ, they can be accurately measured using MEC-type sensors. Interestingly, throughout the experiment, using Ag+ as the toxicant regardless of whether the sensor was encapsulated with PDA, the maximum *IR* obtained was only 47.4 ± 0.2% (PDA-encapsulated sensor, 300 ppm). Experiments using Cu^2+^ and Fe^3+^ as the toxicants yielded maximum *IR* values exceeding 80%. Although this does not affect the accuracy of the sensors in detecting toxicant concentrations, the varying toxicity levels of different toxicants are meaningful research topics that will be further explored in subsequent studies.

Furthermore, the *LOD* and *EC50* for detecting the three heavy metal ions by the sensors with and without PDA encapsulation were determined through analysis and calculation of standard curves. Briefly, after the PDA encapsulation, the *EC50* of the three heavy metal ions increased, indicating that the PDA-encapsulated sensor can be used to detect a broader range of toxicant concentrations. However, except for Fe^3+^, the *LOD* for the other two heavy metal ions also increased, suggesting that the PDA-encapsulated sensor is unsuitable for detecting lower concentrations of the toxicants. The increase in *EC50* and *LOD* is attributed to the protective effect of PDA on the electroactive biofilm.

**Figure 4:**
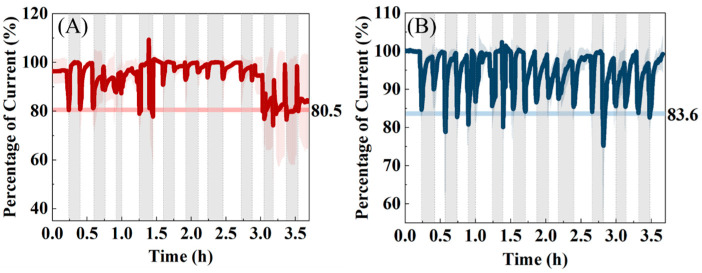
Current vs. time plots for twenty 30 ppm Cu^2+^ consecutive toxicity tests performed on the control (**A**) and PDA-encapsulated (**B**) sensors. The horizontal line represents the result calculated from the standard curve.

After adding a toxicant to the sensor, the current returns to a stable state within a certain period (Figure 2). Building on this observation, a series of twenty consecutive toxicity experiments were conducted using a 30 ppm Cu^2+^ solution as the toxicant. The time-current curves were normalized, meaning that after adding the toxicant, the sensor current was allowed to stabilize without changing the culture medium. Subsequently, the toxicant was repeatedly added again to assess the sensor’s ability for continuous detection. The experimental results indicate that the sensor without PDA encapsulation exhibited good detection results only in the first three tests (Figure 4A), which closely align with the 80.5% obtained through calculations based on the standard curve. This process demonstrates the sensor’s performance in continuous detection after exposure to the toxicant and highlights the impact of PDA encapsulation on the sensor’s detection capabilities. The results obtained in the seventh and eighth tests, with 78.9 ± 7.5% and 77.8 ± 19.3%, respectively, indicate inconsistent detection. Despite achieving results close to the expected value in these tests, the inaccurate readings from the fourth to sixth consecutive tests suggest that the sensor without PDA encapsulation is unsuitable for continuous toxicant detection. In the ninth to sixteenth toxicity experiments, the current percentage obtained was significantly higher than the expected value of 80.5%. During this phase, the sensor qualitatively responded to the toxicant impact but could no longer accurately reflect the toxicity level and concentration. Subsequently, in the seventeenth to twentieth toxicity experiments, the current percentage returned to around the calculated value of 80.5%. However, in the final less than one hour, the standard deviation of the current percentage remained around 20% for the repeated experiments, reaching as high as 23.6%. Furthermore, the current fluctuations over time are not limited to the moments when the toxicant was added, indicating that the sensor had completely lost its ability to detect the toxicant, and the parallelism between the different sensors could not be guaranteed.

In contrast, the PDA-encapsulated sensor demonstrated superior performance in the continuous toxicity experiments (Figure 4B). Throughout the twenty consecutive toxicity experiments, only two experimental values (90.0 ± 4.8% in the second test and 75.2 ± 14.4% in the sixteenth test) fall outside the fluctuation range of the expected value of 83.6% ± 5%. The results of the remaining eighteen experiments range between 78.6% and 88.6%, which meet the accuracy requirements of the experiment. From an error perspective, among the eighteen valid experimental results, except for the third test with 78.8 ± 15.6%, the standard deviation of each other toxicity experiment is below 6%. Furthermore, the standard deviations of the toxicity results from two consecutive toxicity experiments, each consisting of twenty tests, were calculated. For the control, the standard deviation reached an astonishing 7.4%, while that of the polydopamine-encapsulated sensor was 3.4%. This calculation included outliers from the twenty consecutive experiments, sufficiently demonstrating that the polydopamine-encapsulated sensor significantly enhances stability in continuous detection. Thus, it can be suggested that the PDA encapsulation not only enhances the sensor’s durability in continuous toxicity determination but also ensures the reproducibility of the results by different sensors. It is worth noting that after completing the twentieth toxicity experiment, the current of the PDA-encapsulated sensor recovers to the pre-experiment level, with the current percentage around 100%. This observation, therefore, indicates that the twenty consecutive toxicity experiments are not the limit for the PDA-encapsulated sensor. In contrast, the control group of sensors without PDA encapsulation was only successful in three consecutive toxicity detection experiments, signifying the protective effect of PDA encapsulation on the sensor.

### 3.3. Mechanism of PDA Protection of the Electroactive Biofilm

**Figure 5:**
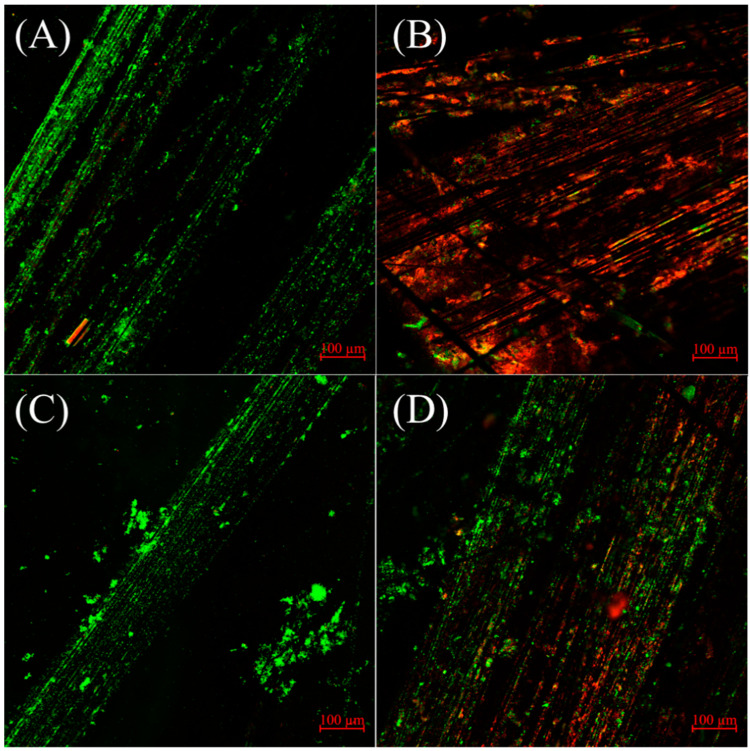
CLSM images of the control and PDA-encapsulated anode electroactive biofilms before and after twenty 30 ppm Cu^2+^ consecutive toxicity tests: (**A**) control group before the experiment, (**B**) control group after the experiment, (**C**) PDA-encapsulated biofilm before the experiment, and (**D**) PDA-encapsulated biofilm after the experiment. The green/red represent Live/Dead bacteria, respectively.

The use of Confocal Laser Scanning Microscopy (CLSM) to capture images of the electroactive biofilms before and after the consecutive toxicity experiments provides qualitative insights. Prior to the toxicity experiments, both sensors with and without PDA encapsulation exhibited highly active green surfaces (Figure 5A,C). After the twenty consecutive toxicity experiments, the biofilms of the control group sensor without PDA coating appeared red (Figure 5B), indicating a significant number of dead cells. This observation aligns with the previous experimental findings (Figure 4), explaining the inability of the control group sensors to conduct accurate detection due to extensive cell death. On the other hand, the PDA-encapsulated biofilm (Figure 5D) largely maintained its original green color despite some areas showing red coloration after the twentieth consecutive toxicity experiment. Thus, it indicates that the electroactive biofilm retained its activity over continuous use, highlighting PDA’s critical protective role. This finding is also consistent with previous research on the protective effects of PDA on electroactive biofilms during the synthesis of nanoparticles [20].

**Figure 6:**
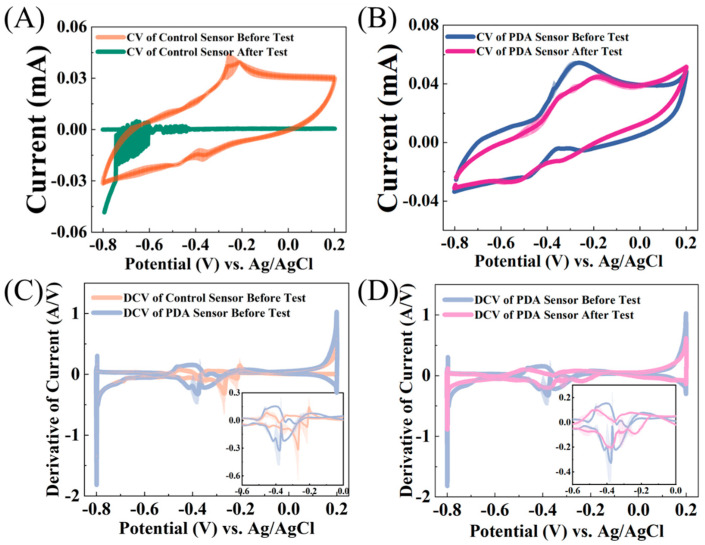
CV of the (**A**) control and PDA-encapsulated sensor (**B**) before and after the consecutive toxicity tests. DCV of the (**C**) control and PDA-encapsulated sensors before and (**D**) the PDA-encapsulated sensor before and after the consecutive toxicity test.

Before and after the consecutive toxicity experiments, the electroactive biofilms were also subjected to cyclic voltammetry (CV) and the first derivative of the CV (DCV) was calculated. A comparison of the CV results of the control group sensors without PDA encapsulation before and after the consecutive toxicity experiments (Figure 6A) clearly shows that the sensor without PDA encapsulation had completely lost its electroactivity after the toxicity experiments. This finding supports the results of CLSM (Figure 5B) and provides an electrochemical perspective on the severe damage suffered by the electroactive biofilm during the seventeenth to twentieth toxicity experiments in the consecutive toxicity study. The inability to accurately feedback the toxicant’s concentration or even the toxicant’s addition can be attributed to the significant damage incurred by the electroactive biofilm, leading to almost complete cell death and the complete loss of electroactivity. On the other hand, the CV of the sensor encapsulated with PDA did not exhibit significant shape changes, with only a slight decrease in the peak oxidation current near −0.3 V, from 0.054 mA to 0.045 mA before and after the consecutive toxicity experiments (Figure 6B). This indicates that the consecutive toxicity experiments did not have a significant impact on the PDA-encapsulated electroactive biofilm. Similarly, the DCV graph (Figure 6D) also demonstrates that the PDA-encapsulated sensor suffered minimal changes before and after the consecutive toxicity experiments. Notably, the oxidation–reduction peaks near -0.36 V nearly overlapped, suggesting that the electroactive biofilm’s oxidation–reduction active substance (possibly a type of cytochrome) remained practically unchanged [28]. However, the absolute value of the DCV peak at this potential after the toxicity experiments 0.48 A/V was lower than before 0.30 A/V, indicating that the consecutive toxic impacts also affected the electroactive PDA-encapsulated biofilm, resulting in a decrease in the extracellular electron transfer capacity of the biofilm.

The protection of PDA encapsulation on the electroactive biofilm in the toxicity experiments can be evaluated by comparing the peaks of DCV in Figure 6C. It can be noted in the figure that the DCV peak has shifted from −0.40 V to −0.48 V after PDA encapsulation, possibly due to dopamine altering the electrode’s chemical properties. However, the absolute change in the DCV peak is insignificant, indicating that PDA encapsulation has not greatly affected the extracellular electron transfer of the electrochemical biofilm. The same is also reflected in the current values during the first 15 min of the current vs. time graph in Figure 2. Therefore, the impact of PDA encapsulation on the electroactive biofilm in terms of electrochemistry is evident in its ability to maintain high electroactivity even after experiencing consecutive toxic shocks.

## 4. Conclusions

In this study, we successfully enhanced the performance of microbial electrochemical biotoxicity sensors via PDA encapsulation. This strategy greatly improved the sensor’s stability and durability in continuous toxicity impact experiments. Although the sensor’s sensitivity slightly decreased, its detection range was significantly enhanced. Furthermore, even after multiple toxicity tests, the sensor’s current could recover close to the initial level, suggesting good reusability. Additionally, the introduction of PDA effectively alleviated the limitations of traditional electrochemical biofilms being susceptible to environmental fluctuations, enhancing the sensor’s long-term operational capability. Confocal laser scanning microscopy (CLSM) and cyclic voltammetry (CV) confirmed that the PDA-encapsulated electroactive biofilm maintained higher activity after toxic impacts despite a slight decrease in extracellular electron transfer capability, demonstrating an overall protective effect. In summary, the PDA encapsulation strategy offers a new perspective for developing efficient and stable microbial electrochemical toxicity detection sensors, providing a powerful tool for future toxicity monitoring and environmental protection.

## Data Availability

The original contributions presented in the study are included in the article, further inquiries can be directed to the corresponding author/s.

## References

[1] Chu N., Liang Q., Hao W., Jiang Y., Liang P., Zeng R.J. (2021). Microbial electrochemical sensor for water biotoxicity monitoring. Chem. Eng. J..

[2] Girotti S., Ferri E.N., Fumo M.G., Maiolini E. (2008). Monitoring of environmental pollutants by bioluminescent bacteria. Anal. Chim. Acta.

[3] Clements P.J., Bolon B., McInnes E., Mukaratirwa S., Scudamore C., Haschek W.M., Rousseaux C.G., Wallig M.A., Bolon B. (2022). Chapter 17—Animal Models in Toxicologic Research: Rodents. Haschek and Rousseaux’s Handbook of Toxicologic Pathology.

[4] Weiner A., Irijalba I., Gallego M., Ibarburu I., Sainz L., Goñi-De-Cerio F., Quevedo C., Muriana A. (2024). Validation of a zebrafish developmental defects assay as a qualified alternative test for its regulatory use following the ICH S5(R3) guideline. Reprod. Toxicol..

[5] Charoeythornkhajhornchai P., Kunjiek T., Chaipayang S., Phosri S. (2023). Toxicity assessment of bioplastics on brine shrimp (*Artemia franciscana*) and cell lines. Emerg. Contam..

[6] Clerbaux L.-A., Coecke S., Lumen A., Kliment T., Worth A.P., Paini A. (2018). Capturing the applicability of in vitro-in silico membrane transporter data in chemical risk assessment and biomedical research. Sci. Total. Environ..

[7] Jin X.-W., Li Z.-Y., Xu P.-P., Zhang X.-Y., Ren N.-Q., Kurilenko V.V., Sun K. (2019). Advances in Microfluidic Biosensors Based on Luminescent Bacteria. Chin. J. Anal. Chem..

[8] Yang M., Zhang H., Yao Y., Lin W., Duan S., Liu B. (2023). Characterization of Light-Sensitive Refractive Indices for Ionic Liquids Based on a Coreless-Fiber-Coupled Microcavity Interferometric Sensor. IEEE Sens. J..

[9] Wang J., Dong C., Li Q., Yang X., Li D., Zhang L., Zhang Y., Zhan G. (2023). Innovative electrochemical biosensor with nitrifying biofilm and nitrite oxidation signal for comprehensive toxicity detection in Tuojiang River. Water Res..

[10] Wu Y., Wang C.-W., Wang D., Wei N. (2021). A Whole-Cell Biosensor for Point-of-Care Detection of Waterborne Bacterial Pathogens. ACS Synth. Biol..

[11] Atkinson J.T., Su L., Zhang X., Bennett G.N., Silberg J.J., Ajo-Franklin C.M. (2022). Real-time bioelectronic sensing of environmental contaminants. Nature.

[12] Adekunle A., Raghavan V., Tartakovsky B. (2019). A comparison of microbial fuel cell and microbial electrolysis cell biosensors for real-time environmental monitoring. Bioelectrochemistry.

[13] Du L., Yan Y., Li T., Liu H., Li N., Wang X. (2022). Machine Learning Enables Quantification of Multiple Toxicants with Microbial Electrochemical Sensors. ACS ES&T Eng..

[14] Li T., Chen F., Zhou Q., Wang X., Liao C., Zhou L., Wan L., An J., Wan Y., Li N. (2020). Unignorable toxicity of formaldehyde on electroactive bacteria in bioelectrochemical systems. Environ. Res..

[15] Luo H., Liu G., Zhang R., Bai Y., Fu S., Hou Y. (2014). Heavy metal recovery combined with H2 production from artificial acid mine drainage using the microbial electrolysis cell. J. Hazard. Mater..

[16] Liu Y., Ai K., Lu L. (2014). Polydopamine and Its Derivative Materials: Synthesis and Promising Applications in Energy, Environmental, and Biomedical Fields. Chem. Rev..

[17] Kim M., Li S., Song Y.E., Park S.Y., Kim H.I., Jae J., Chung I., Kim J.R. (2023). Polydopamine/polypyrrole-encapsulation graphite felt enhances biocompatibility for electroactive bacteria and power density of microbial fuel cell. Chemosphere.

[18] Du Q., Li T., Li N., Wang X. (2017). Protection of Electroactive Biofilm from Extreme Acid Shock by Polydopamine Encapsulation. Environ. Sci. Technol. Lett..

[19] Du Q., Mu Q., Cheng T., Li N., Wang X. (2018). Real-Time Imaging Revealed That Exoelectrogens from Wastewater Are Selected at the Center of a Gradient Electric Field. Environ. Sci. Technol..

[20] Liu Y., Zhu X., Zhao Q., Yan X., Du Q., Li N., Liao C., Wang X. (2021). Synthesis of silver nanoparticles using living electroactive biofilm protected by polydopamine. iScience.

[21] Liu Y., Zhao Q., Liao C., Tian L., Yan X., Li N., Wang X. (2023). Anaerobic bioreduction of elemental sulfur improves bioavailability of Fe (III) oxides for bioremediation. Sci. Total. Environ..

[22] Wang J., Chen M., Zhang J., Sun X., Li N., Wang X. (2024). Dynamic membrane filtration accelerates electroactive biofilms in bioelectrochemical systems. Environ. Sci. Ecotechnol..

[23] Sheng G., Yu H., Li X. (2010). Extracellular polymeric substances (EPS) of microbial aggregates in biological wastewater treatment systems: A review. Biotechnol. Adv..

[24] de Menezes A., Clipson N., Doyle E. (2012). Comparative metatranscriptomics reveals widespread community responses during phenanthrene degradation in soil. Environ. Microbiol..

[25] Wang X., Gao N., Zhou Q. (2013). Concentration responses of toxicity sensor with Shewanella oneidensis MR-1 growing in bioelectrochemical systems. Biosens. Bioelectron..

[26] Xu M., Li J., Liu B., Yang C., Hou H., Hu J., Yang J., Xiao K., Liang S., Wang D. (2021). The evaluation of long term performance of microbial fuel cell based Pb toxicity shock sensor. Chemosphere.

[27] Light S.H., Su L., Rivera-Lugo R., Cornejo J.A., Louie A., Iavarone A.T., Ajo-Franklin C.M., Portnoy D.A. (2018). A flavin-based extracellular electron transfer mechanism in diverse Gram-positive bacteria. Nature.

[28] Su H., Yan X., Zhao Q., Liao C., Tian L., Wang Z., Wan Y., Li N., Wang X. (2023). Layered Design of a Highly Repeatable Electroactive Biofilm for a Standardized Biochemical Oxygen Demand Sensor. ACS Sens..

